# Practitioner perspectives on working with older patients in opioid agonist treatment (OAT) in Norway: opportunities and challenges

**DOI:** 10.1186/s13722-024-00473-7

**Published:** 2024-05-23

**Authors:** John Todd-Kvam, Thomas Clausen

**Affiliations:** 1 Norwegian Centre for Addiction Research, University of Oslo, Blindern, Postboks 1039, 0315 Oslo, Norway; 2 Sørlandet Sykehus HF, Lundsiden, Postboks 416, 4604 Kristiansand, Norway

**Keywords:** Opioid agonist treatment, Quality of life, Ageing, Practitioner perspectives

## Abstract

**Background:**

Norway has a growing proportion of ageing opioid agonist treatment (OAT) patients, with 42% of the 8300 Norwegian OAT patients aged over 50 in 2022. This study aims to explore practitioners’ views and experiences from treatment of ageing OAT patients.

**Methods:**

Data were collected as a series of semi-structured interviews with treatment staff (roles interviewed: doctor, psychologist, social worker, nurse, and learning disability nurse). Participants were recruited from three OAT outpatient clinics, one with an urban catchment area and two with a mix of urban and rural. The interviews incorporated questions on patients’ somatic and mental health, strengths and weaknesses of the service for this group, and patients’ quality of life.

**Results:**

Older patients were perceived to be more often stable in terms of substance use and housing situation, but also experiencing some key challenges in terms of cognitive impairment, loneliness and isolation, and comorbidities. Both the practitioner-patient relationship and healthcare interactions outside OAT had the potential to impact treatment quality positively or negatively depending on how they were managed.

**Conclusions:**

Treating older patients in a way that respects and enhances their dignity is important. We argue that this requires better services for those whose functioning is impacted by cognitive impairment/dementia, an age-informed treatment model for this patient group, along with urgent work to improve municipal-level services given practitioners describe them as unacceptable in certain areas.

## Background

Opioid Agonist Treatment (OAT) has prolonged lives of patients^1^ compared to those not in treatment, to the extent that over 42% of Norwegian OAT patients are now over 50 and the average age of those in treatment is 47.8, up from approximately 38 years when the program started 25 years ago [29]. Previous research has shown a high retention rate compared with other programs, at 66% in continuous treatment 18 months after treatment inclusion [5], and the program has a high coverage rate with about 80% of the target population in treatment [12]. Overall, these figures speaks to the OAT population who enter treatment stay longer, age, and experience a much lower overdose mortality [8] and lower all-cause mortality [35] than outside OAT, and hence become an ageing population in treatment. OAT patients living longer is a success story, albeit one that creates new needs in terms of research knowledge and its clinical application. We need to know more about these patients’ needs and the healthcare system’s capacity to assess and meet these needs. This article aims to contribute to this by shedding light on how practitioners working within the specialised health service assess the life-situations of older OAT patients and the quality of treatment they receive.

### Research on OAT and ageing

Two recent reviews have identified a dearth of studies examining tailored treatment services for opioid use in older adults/older OAT patients [6, 42]. There have also been calls for research on the specific and unique health needs of the growing population of older OAT patients [18], with ([10], p. 1) arguing that “Many unanswered questions on medical, psychosocial and health economic consequences remain as the needs of older patients have not yet been evaluated extensively.” Roe et al. [32] call for more research on the specific needs of an ageing population, whilst there have also been calls for research on staff attitudes at treatment centres [24], p. 1344), on comorbidities and early interventions amongst an ageing patient population [13] and on mental health issues among older people engaging in substance use [37]. There has also been a call for research on multiple chronic disease management and how to maintain physical and cognitive function for middle-aged and older adults with opioid dependence [18].

Regarding what we do know, there have been quite different conclusions drawn on ageing OAT patients: some give grounds for optimism whilst others raise major challenges. For example, a US study concluded (against authors’ expectations) that older methadone treatment patients may, in fact, have fewer problems and succeed in treatment—albeit with a caveat that more research is needed to confirm the findings [15]: 541). In contrast meanwhile, ([34] pp. 494–495) observed that those participants who were ‘young’ older adults (50–55) were in fact closer to the end of their lives than to middle age. The authors highlight an urgent need for substance use services to meet the geriatric service needs of this group. In terms of mental health disorders, it has been argued that these may be exacerbated by age-related comorbidities and social isolation [21]. There are also indications that accessing services becomes more difficult with age, due in part to experienced stigma, to a sense of shame at still needing support even at an older age and to unwillingness to associate with younger, potentially more chaotic people who use drugs [1, 9, 25].

Regarding what we know about Norway, ageing OAT patients experience a large range of somatic disease burdens [27],see also [31], with high prevalence of smoking and low rates of cessation [4]. Indeed, neoplasms are recorded as the leading non-overdose causes of death amongst patients over 50 [11]. Older patients also report facing stigmatization, isolation and financial difficulties [7].

Overall, we have a complex picture of some positive observations regarding success in treatment, but with extra challenges including experiencing age-related issues early, social isolation and difficulty accessing services. There are also significant research gaps both regarding treatment of older OAT patients in Norway and internationally. Our analysis below aims to fill in some of this territory by analysing how OAT practitioners perceive the treatment older patients receive both in OAT and more broadly.

### How OAT works in Norway

OAT in Norway is designed as a three-way cooperation between specialist treatment services, municipal-level health and social services and primary care doctors/GPs. Through this cooperation, OAT in Norway aims to provide:

- Assistance with basic living conditions such as housing, finances, activities, education, employment, and family/network.
- Treatment in interdisciplinary specialized treatment (hereafter referred to as specialist OAT).
- Detoxification if desired and needed.
- Assessment and treatment for somatic and mental health issues if desired and needed.

Whilst originally introduced as a high-threshold service with strict inclusion criteria, OAT in Norway is now a low-threshold service that should offer a flexible approach to different patients based on their treatment needs and life situations. Historically, the programme was rehabilitation and total abstinence oriented, while now it also includes “low-threshold OAT”, which means ongoing substance use is to some extent accepted while on OAT medications, as long as the overall treatment is perceived as better than being off treatment. For some, treatment might involve a very light-touch approach with only annual check-in meetings with specialist OAT and for others it can mean daily supervised intake of medicines and regular follow-up and focus on control/medical safety in treatment (urine testing and other measures designed to ensure patient safety and prevent diversion are labelled ‘control/safety measures’ given that they have an in-built duality of both intention and experience). Overall, current Norwegian OAT aims to support different treatment goals, be it rehabilitation or stabilisation and harm-reduction, with about 70% of patients stating they have a “rehabilitation goal”, while 30% state they have a low-threshold and harm-reduction oriented goal for their treatment [29].

At the end of 2022, there were approximately 8300 people in OAT in Norway. Of these, nearly 80% have their treatment anchored in specialist OAT, whilst municipal-level services (including GPs) have been delegated responsibility for the remainder of the patient group whose treatment location is known [29], p. 25). There is though significant geographical variation under these headline figures, with 98.3% of patients primarily followed up in specialist OAT in Bergen and only around 15% in Asker/Bærum, a region just west of Oslo. There are also large variations in prescribing practice, with methadone accounting for less than 10% of prescriptions in one region and over 50% in others [29], p. 15). Just under 40% of OAT patients are also prescribed benzodiazepines (again, this varies regionally from less than 20% to 60%) [29], p. 18). Individual dispensing arrangements for OAT medicines is, according to the treatment guidelines, to be “based on an assessment of the patient's substance use, treatment and rehabilitation needs, and the risk of the medication becoming available to third parties” [20], p. 88), with patients collecting on average three times per week [29], p. 22). Use of urine testing has decreased over time, with those subject to regular testing reducing from 43% in 2019 to 25% in 2022. Interestingly, two adjoining municipalities in the Oslo region have the largest variation here, with nearly 90% of patients experiencing sporadic/regular urine testing in one whilst only around 45% do so in the other [29], pp. 23–24). Variation in service provision is also an important topic in our analysis below.

## Methods

### Aim

This article flows from a larger study aiming to improve understanding of ageing, substance use and OUD with the goal of improving treatment and quality of life. Specifically, our aim with this article is to shed light on how practitioners working within the specialised health service assess the life-situations of older OAT patients and the quality of treatment they receive. Through a fine-grained qualitative approach (i.e. placing our focus towards the 'smaller' end of Bamberg and Georgakopoulou [3]’s spectrum from micro analysis to macro accounts), we hope to stay close to our participants’ experiences of working with a multiply-marginalised patient group who have faced—and continue to face—challenges across a range of domains.

## Data collection

Practitioners (N = 10) were purposively recruited from three hospital trusts, one with a purely urban catchment area and two with an urban/rural mix. Two of the trusts were content with an informal approach via email, whilst one requested a formal application (which was quickly approved). Geographic variation in service provision has previously been highlighted in Norway, so recruiting from more than one area was important to capture some of this variation. We also wanted to capture a range of different professional backgrounds, and as such interviewed people with the following roles: social worker, senior consultant, clinical psychologist, learning disability nurse/social educator, and nurse. Interviews lasted 30–60 min and were conducted over Zoom. All participants provided informed consent.

Interviewing via videoconferencing was necessary given the interviews took place during Covid-19 restrictions. Whilst in this specific case it was a condition for conducting the study, it also had the advantage of lowering the threshold for participation (participants were healthcare workers under extra pressure during the pandemic). Virtual interviewing does though mean it is more difficult to build rapport and respond to body language cues during the interview [22]. The first author’s experience was that when participants displayed affect (e.g. frustration, anger, or pride), it was possible to pick up on this despite not being in the same physical space. More subtle cues, though, may well have been missed.

The range in interview length can in part be explained by context—all aspects of the healthcare system were under greater pressure during the pandemic, so some staff only had capacity for a shorter interview. Having a semi-structured approach was useful here in that it was possible to address the most important topics even in a limited timeframe. The interviews covered the following topics from the perspective of those over the age of 50 in OAT:

- Practitioners’ role and place in the treatment apparatus
- What works well/badly in OAT, and suggested system improvements
- How practitioners would describe patients’ quality of life, physical and mental health
- If/how practitioners experience a tension between the control/safety measures and care functions in their roles
- Relationship-building with patients
- What challenges do patients typically face?
- What resources are available to patients?
- If they have noticed changes over time

### Coding and analysis

An initial set of codes was created by analysing relevant literature on OAT and ageing in NVivo. These codes were then used to deductively analyse the practitioner interview transcripts (coding conducted by author one). The codes were refined through this process, with the addition for example of a specific code on stability, which we identified as a theme in our empirical data rather than the literature. When seeking to hybridise these deductive and inductive approaches (see [14], we were informed by Interpretive Phenomenological Analysis [36], in that we wanted to focus on the understandings of our participants and the meanings they narrated as important regarding their experiences of treatment of older OAT patients.

In categorising our findings, we initially aimed to present key opportunities and challenges as described by our participants. That is to say, these are opportunities and challenges as we interpret them *from the perspective of the practitioners.* However, in working with the data it became apparent that it would make sense to include a third category to shed light on issues that, if handled well, provide an opportunity but that if handled badly become a challenge for patients in terms of their treatment and quality of life. Within these overarching categories, we identified themes that participants emphasised as of particular importance for patients in terms of their treatment and quality of life.

### Ethics

The overarching project received ethical approval from the Regional Committee for Medical and Health Research Ethics South East C (ref. 28,848). Data protection approval was granted by SIKT, the Norwegian Agency for Shared Services in Education and Research (ref. 420282). Any identifiable details in the data material have been edited to preserve participant anonymity. Given the small sample drawn from relatively small units, we elected not to present demographic information.

Regarding relational ethics in the interview situation, author one employed what Neumann and Neumann [30] term a ‘confluent’ interaction style in the interview, which prioritises rapport and a positive experience for the participants, whilst having the potential disadvantage of avoiding confrontational questions. There was one specific issue that occurred in a number of the interviews that required careful real-time consideration of relational ethics. Author one’s interpretation of the situation was that there had been an incident with a specific patient and a specific municipality that had severely provoked a number of the participants from one of the hospital trusts (see "Opportunities/challenges" section below). These participants all appeared to express strong anger and indignation on behalf of a patient that came across even via the screen. The first author’s curiosity was piqued by this, but decided it was ethically important not to probe for specific details given that it would risk encroaching on the participants’ duty of confidentiality (and would contradict the confluent interaction style).

## Results and analysis

As stated above, our findings are presented across three main categories: opportunities, challenges, and opportunities/challenges. These categories are based on our interpretation of what the practitioners themselves saw as opportunities, challenges, or both. Patients themselves may well take a different view (see [40] for more on the patient perspective).

### Opportunities

Two, rather interlinked, opportunities that practitioners discussed where of having achieved a degree of stability, both in terms of lifestyle and substance use and in terms of accommodation.

#### Stability—less substance use, calmer lifestyle

Regarding a more stable lifestyle, practitioners attributed this to two factors, the first being getting tired of or ‘ageing out’ of substance use:

Yes, those who are 50+ are, at least - now I can only speak from the perspective of my patients, but they, the older they are, the more stable - or stable, but anyway, they can't bear to live that drug-life anymore. So that there is less side-use and more stability... [Participant 1, P1]

There are many who have tried a lot - many who may be more aware of what works, what doesn't work. […] So, at least some of them in this group have actually started to get tired of the drugs and maybe the party stopped a long time ago, and well, it's not as hectic - it's more calm around them, things have settled, people have - well, there are some who are doing well and living drug-free and are fine, and others who are actively using drugs in a way, but still, it's maybe more peaceful around them. [P2]

We can see here practitioners observing older patients as being more ‘stable’, ‘settled’ and that they ‘can’t bear’ the more chaotic lifestyle they had before. These observations chime with Carlsen et al. [7]’s findings regarding more stable life situations amongst older OAT patients in Norway, as well as Firoz and Carlson [15]’s findings from the US. The second factor practitioners identified was of patients coming to terms with OAT and its influence on how they lived their lives:

Otherwise, all of mine are those who have been here for a long time or they come in for the second, third, or fourth time, so they know the system in a way. It's calmer for them, and they tolerate the control aspect much better. Some of them may even express that they like it when we say "no, not now" and set up more frequent collection arrangements. They can see some form of care in it, rather than exhibiting aggression and anger, at least mostly. Of course, there are exceptions, but it seems to be calmer in that regard. [P3]

We can see here how this practitioner sees care being expressed through control/safety measures (see also [38] on this). Whilst we explore in "Opportunities/challenges" section below how control/safety measures can also be experienced negatively, here practitioners relate that, for some older patients, their coming to terms with life in OAT can be positive in terms of stability and ‘calmer’ interactions.

#### Living accommodation

In general, practitioners reported that older patients have had stable living accommodation over some time:

The vast majority have their own home, or they all have their own home, but there are two who live in what we would call a housing community. So, for most people it has been a home they have had for a long time. [P3]

Yes, as I said, I believe most of them have settled down quite a bit. There are very few who don’t have housing, and it seems like things have improved for most of them, that’s my impression. I can’t say that I have personally experienced it [housing] as a specific challenge related to age. [P4]

Given that we know that unstable housing and homelessness impact on quality of life and mortality [17], these reports of stable living accommodation are positive. Survey data would align with these observations, with 84% of those over 50 in OAT reporting having their own living accommodation (as opposed to, for example, hospice/hostel, homeless or in prison); this figure was 62% and 75% for those under 30 and between 30–50 respectively [41], pp. 9–10). There were though some concerns expressed about the availability of supported accommodation as OAT patients age:

It would be ideal if we could have ongoing social housing support for this group that lasts a lifetime. We can see that many, even in this group, struggle to live in adequate conditions. They are content with living poorly, but they struggle to tidy up and maintain order. This also means that relatives and others avoid visiting them, there might be unpleasant odours, and overall, many of them have subpar living conditions. It would be beneficial to have services that take ownership of this issue and ensure that their living conditions are dignified, meaning clean, tidy, and a pleasant place for everyone to visit. […] I see that some individuals end up living a more undignified life than they deserve, in my opinion. This is because I have a few cases where individuals create such a mess in their apartments that people eventually stop visiting them, leading to feelings of loneliness, and they don’t see the connection. [P3]

I have several patients, and I made a list of the patients I have who are over 50. Many of them have faced challenges in finding suitable housing because their needs have become quite complex, and they may require more assistance related to housing since they are not able to manage everything on their own. […] We can have meetings with a multidisciplinary team, where we have a long list of actions and tasks that should be carried out, but month after month, I see that these things are not being followed up on, and it creates a lot of frustration. So, housing and overall support are challenging because these patients may require quite a lot, and perhaps the city-district does not have the resources to meet their needs adequately. The staff at the welfare office are overwhelmed with sick leave and overwork, and I understand that it is demanding, but there is a need for improvement in this area. [P5]

Both of these excerpts highlight how interconnected and complex these issues can be. Practitioners describe experiences of patients who struggle to live up to norms of cleanliness and tidiness end up unwittingly driving away friends and family, leading to isolation (and, most likely, worsened standards of self-care as a result). Also, how the need for supported accommodation requires cooperation with other elements of the welfare apparatus whose resourcing and/or culture create long waiting times and frustrations for patients and their contacts in OAT. This latter point reinforces Gaulen et al. [16]’s findings on lack of supported housing in high demand areas in Norway.

A final, rather bittersweet, story was told by one practitioner. The story encompasses a number of themes relevant to this patient group, including somatic health problems and early mortality. It is though at its core a story about providing dignity through appropriate accommodation at the end of life:

I remember one—this is a positive one—although he has passed away now, died of cancer. But the first time I visited him after he had been transferred from the hospital to respite care at the nursing home—when I first visited him—he was clean and smelled good. It was like, he had been living in an institution, a staffed residence, yet he was really capable of taking care of himself. But it was just so, it was a really nice, wonderful thing. I felt that they had truly—now they had given him, he felt like a king, you know (laughter). Previously, he had never felt that he deserved to receive any kind of support or services, but now he was so—so happy, so content. He felt he had had a good life—he was, how old was he, yeah, I think he was 53 or 54 when he died of cancer. [P6]

An important element of this story is the symbolic impact of being provided with good quality care. The practitioner relates how the patient did not feel worthy of support before but was happy and content in a nursing home even whilst terminally ill with cancer. In this case, there was an opportunity to provide someone with dignity and respect through good quality care. Overall, housing was in general seen as a positive, albeit that practitioners describe potential for both positively and negatively reinforcing processes depending on the availability of stable and appropriately supportive living accommodation as patients age.

### Challenges

Practitioners identified a range of challenges facing older OAT patients. We interpreted practitioners as seeing three challenges as particularly important: (1) problems with cognitive impairment, (2) loneliness/isolation and (3) comorbidities.

#### Problems with cognitive impairment

Severe cognitive impairment amongst older people accessing OAT has been observed internationally [2, 23, 33]. Practitioners also raised this as a challenge with a range of implications, with the most acute being accessing services and support outside specialist OAT:

[S]ome people have a need for a lot of primary services, such as support contacts, home care nurses, someone who cleans their home, someone who is just there and looks after them, takes them out a bit, you know. I wish there were maybe a bit more of those available for some of the patients we've had. It becomes a bit challenging when things start to, you know, if you're an OAT patient who has been actively using drugs on and off, maybe starting to become forgetful, not remembering things very well. There aren't many places we can turn to in terms of where these patients can be placed. So I wish there were more options out there in terms of both primary services and treatment or care facilities. [P7]

The practitioner here describes experiencing a general problem regarding inadequate structural-level provision for OAT patients experiencing cognitive impairment. Specific problems were also highlighted:

There are also some patients who have dementia at a fairly early age, who get typical old-age ailments way before their time. To begin to gain an understanding that even if you are not yet 60, a nursing home is the future. We have to make the municipality understand that now they have to come in with help. We have something totally, totally, it's so grotesque you know, you won't believe it, I have an example now that I work with, not one of my patients, then I would have gone crazy I think, but it's so grotesque then the way the municipality meets an OAT patient who very likely has dementia, erm, that if it had become public, then people would have had to resign from their positions, it's absolutely horrible. And it's such a frustration. [P3]

Yes, we experience that quite a lot, that we receive patients who we start to wonder if they are developing dementia or where we are unsure about it - yeah, that they have reduced cognition. It is very important with good collaboration with the municipality and the primary health care service, GPs, home nurses. There we are experiencing major challenges with a number of municipalities, that they have a very principled approach to OAT, that they do not want to distribute OAT medication. […] And this is a challenge that may become more visible now that more people need home nursing care. [P4]

These accounts include stark and specific criticism of some municipalities and their attitude to vulnerable OAT patients, as well as a more overarching observation about a deficit of appropriate care and treatment facilities. In terms of the specific criticisms of certain municipalities, this was a theme from one hospital trust in particular, whose catchment area included some small, rural municipalities. Practitioners described attitudes and care-provision in these municipalities as ‘grotesque’, ‘grave’ and, as discussed in "Opportunities/challenges" section below, ‘jaw-dropping’. Whilst we only have the practitioners’ perspective here, those interviewed displayed such indignation and strong affect when discussing this issue that we interpret their concerns as credible.

There was also some comment on how specialist OAT itself has room for improvement with patients who are cognitively impaired:

Yes, right, and we don't really know, is it in a way more like psychological, psychotic things that they get, or is it actually what I call holes in the brain, that something purely cognitive is starting to happen due to prolonged intoxication and poor nutrition and things like that. Encountering something like that means stopping and thinking a little "What could this be about?" and "How on earth are we going to be able to figure it out" I think it's a bit like that, at least from my perspective as a psychologist, it is often important to take that step back to see "Why don't they turn up? What is it that happening? Why are they getting so massively drunk now?" And that the system can sometimes - we can get a little upset. So if I were to think of something that I would like that we could maybe do better - I don't have a recipe, but I think that we could look at this. [P8]

Here we can see an acknowledgement that specialist OAT could usefully make room for more reflection about the underlying causes of patients not turning up to appointments, excess alcohol use or other behaviours that have the potential to negatively impact the practitioner-patient relationship. Overall, the issue of identifying cognitive impairment and providing appropriate care is already a major challenge and is likely to become even more pressing as this cohort of OAT patients ages further. Care must be taken to provide the appropriate type of treatment and support that respects patients’ ambitions and functioning. That practitioners asserted that some small municipalities are unwilling/unable to provide appropriate support is of concern and needs to be researched further.

#### Loneliness/isolation

Practitioners reported that older patients experienced loneliness and isolation:

I think perhaps the most common obstacles are that they leave themselves a bit to themselves, they become socially handicapped in a way, then they sit at home and think "that's what my life is now", eh their loneliness, they break a little too rarely out of that loneliness. [P3]

So, there is less substance use and more stability, but at the same time, I find that many of them say that they are lonely. Because they can't be with the drug-using community they were part of before, and they haven't been able to establish a drug-free network, so they are alone. [P1]

The first of these excerpts places a fairly high degree responsibility on patients themselves to ‘break out’ of their loneliness. We also consider that the expression ‘socially handicapped’ to be unfortunate, not least in terms of how it juxtaposes with the just-mentioned responsibilisation. The second speaks more to the social context in which older patients find themselves and the difficulties of building drug-free networks/new relationships (see also [7]. Separate interviews with older patients also emphasised this challenge, so this is a shared concern that impacts on quality of life [28].

Practitioners also discussed how their own long-term relationships with patients could help to alleviate isolation in some ways:

Here's one person I call twice a year, at the top of my list here, and we talk about fishing. He invites me up every year, saying, "You must come up soon and go fishing," […] So we can also talk about the boat, and I visit his home once or twice a year. […] And we just sit and I listen to stories about the cats in the house and the neighbours he's struggling with. His vision is incredibly poor, but he still enjoys his stamp collection, coin collection, and all these little things that shape his daily life. We touch upon topics like children, grandchildren, and it becomes more sensitive again, as he doesn't have much contact with them. So that feeling of loneliness, which many OAT patients experience, almost regardless of age, it intensifies for some of these older individuals. I have several of them who feel very alone, um, and I believe that receiving a phone call from time to time and knowing they can do the same means a lot to them. [P3]

This account has several facets. On one reading, this a positive story of how a long-term and trusting relationship between practitioner and patient can alleviate loneliness. On another, it is unfortunate that patients appear to rely to such a degree on their contacts in OAT and have little social network aside from those exercising a professional role. Research with people who have experience of both recovery from addiction and desistance from crime^2^ in Norway has identified a similar issue, with participants wondering when they will achieve ‘normality’ without all their social interactions being with some form of institutionalised help or welfare [39].

#### Comorbidities

Comorbidities—and in particular somatic comorbidities—were emphasised as a challenge by practitioners, both in and of themselves:

For many their somatic health deteriorates over time, well, in many ways actually. Teeth are a big problem, very poor dental health. And I've also had this issue with, well, they develop things a bit earlier than others, like liver and kidney failure, there are quite a few problems there, even though most of them have received treatment for hepatitis C, and there have been some who have died from multiorgan failure without having active hepatitis C. Cancer is a common cause of death. Chronic obstructive pulmonary disease, especially. Yes, so they actually experience common somatic disorders, but they may experience them a bit earlier and become very ill earlier. At the same time, I see that many live with it for a long time as well. [P6]

…and as a problem for managing concurrently with OAT medicines:

But another thing is that we have little experience with the elderly and drug use, we have little - in other words, the knowledge base for that group is a bit poor, so we have a little less knowledge about how the drugs work pharmacokinetically, pharmacodynamically then. And I also think it's difficult, in light of that, to discuss with them about tapering down the dose, for example, or comorbidity or increased overdose - they have an increasing risk of overdose the older they get. [P4]

The narrative of OAT patients experiencing problems earlier was a common theme, and separate interviews with older patients included reference to taking many different prescription medicines daily and an experience of them cancelling each other out. Pain management was also viewed as a problem:

*And what about, those who are 50*+*, when you think about physical health?*

Mhm, well, yes, that too is a very wide range. Um, but I see that we have several who have a lot of somatic issues as well, yes. Who are in the hospital a lot, yes.

*Yes, and what is your impression of the follow-up they receive for these problems?*

Well, um, especially when it comes to pain issues, I find it difficult to get them properly assessed, both because it's challenging in combination with, for example, methadone treatment, but also because there's a bit of, um, yes, maybe there are some prejudices that they come here because they want, um, yes, they want tablets and such. So it has been a bit challenging to get pain assessment and treatment for pain, actually, yeah. [P1]

[T]here have been glaring examples of after surgeries being told, "no, you don't get anything because you're in OAT," well, that’s unacceptable anywhere, right. Without them contacting us, you know, that's the thing, they use us, they use OAT, I almost said, as an excuse, which we don't stand for, that's just wrong. [P6]

These excerpts describe difficulties with pain management as being a combination of a medical challenge (how best to provide pain relief for people on methadone or buprenorphine) and a cultural challenge in terms of meeting prejudice from other healthcare workers (practitioners also describe encountering negative attitudes elsewhere in the system—see "Opportunities/challenges" section below for more on this). Whilst there was also acknowledgement that cooperation and attitudes had improved following efforts from OAT staff, older OAT patients in Norway have lived through a period when attitudes towards them were suspicious and even punitive. Experiences of differentiation and inadequate pain relief are not something easily forgotten and will in many cases be carried with people into new interactions with the healthcare system.

### Opportunities/challenges

Two key issues raised by practitioners that were double-edged were the relationship between specialist OAT and the patient and interactions with other elements of the system outside specialist OAT.

#### Relationships with OAT

Practitioners emphasised the importance of constructive relationships with their patients. As one psychologist remarked:

I sort of try to avoid becoming part of controlling and bothering people, but rather be on the service side. It's also a bit like that I can have my projects, but what are the patient's projects? Don't take it for granted. So, it's maybe a bit of a buzzword, but dignity is actually a very important concept for me when dealing with patients who often have a lot of shame. Who feel that people sit and watch them on the tram, they have to get off because they get scared. So that is, come across as accepting […] I'm becoming a bit like, not privately, but that I can share a bit about common human things, which I think is relationship-building in many cases. [P8]

This excerpt includes reference to several important concepts for relationship-building in OAT in general and with older patients more specifically. The participant discusses a conscious effort to avoid ‘controlling and bothering’ patients and an awareness that patients have their own projects that may not necessarily align neatly with practitioners’ projects for the patients. Regarding older patients, the notion of dignity is in our view of particular salience. As this participant highlights, experiencing significant shame impacts negatively on quality of life (a specific example here is not being able to complete a tram journey because of fear of how others see them, but shame impacts in many ways). The participant describes how an accepting attitude, personal reciprocity (sharing ‘common human things’) and humour can help in managing treatment relationships with vulnerable, often multiply-marginalised people. This personal reciprocity has also been emphasised as important in relationship-building by Norwegian probation caseworkers [38].

Another theme raised by practitioners was continuity and long-term relationships. For example:

Well, regarding continuity, I think that has been very beneficial for many of these individuals. We become a kind of security for them, a place they can turn to. […] Being able to maintain these relationships over time also contributes to making them feel secure. Because, obviously, if there are frequent changes of therapists, they will constantly test the new boundaries, right? But when they have long-term relationships, they probably experience more care, meaning that the control function is also caring. [P6]

We've been pretty stable in the employee group that works with OAT, at least as long as I've been here. Well, you start to get - you've met all the patients several times. And they get the same from us every time, eh, before it was a bit random who they met, I think. [P1]

The first practitioner here links continuity to a sense of security for patients and to more straightforward exercise of OAT’s control/safety functions, affirming that, in a long-term treatment relationship, control can be experienced as caring. This chimes with discussions in the sub section "Opportunities" above but stands somewhat in contrast to excerpts on the negative aspects of control later in this subsection. Before turning to these more negative aspects, there are other facets of the long-term relationship to discuss:

I see here how well some treatment personnel connect with very demanding patients, and I think the contact you have built up over time is very important. [P2]

Yes, and many of these relationships have actually taken years to develop. If you think about it, for many of them, I believe it has been very important because I feel that for some, I may be the person they have had the longest relationship with, you know. I mean, they have had many disruptions in their upbringing, being placed in orphanages and foster homes and such, separated from their siblings, having many different caregivers. So suddenly they have had a relationship with someone who has actually endured some hardships, you know. I actually think that has been important too, and that we don't keep changing even when things get difficult. [P6]

Practitioners here emphasise how patients can lack continuity in their relationships, and that this can begin in childhood. This is important to bear in mind regarding the challenges some in OAT can face in terms of adverse experiences that begin early in childhood. That OAT can provide some experience of continuity is described as positive, giving both patients and practitioners a chance to look back and reflect over their time in OAT. As discussed in the section on loneliness and isolation, some practitioners describe themselves as helping alleviate such experiences though regular contact over time. Separate interviews with older patients have, though, raised concerns that constant staff turnover is a problem [40], so these long-term treatment relationships are not universal. Significant time in OAT can also mean significant experience of its control/medical safety functions. In part this is seen by practitioners as a cohort effect, in that older OAT patients have long experience of Norwegian OAT when it had a much stronger control element:

It's very difficult, you know. (short pause) Because they have - I believe historically they have been exposed to - and maybe it's more those who have been in OAT for a long time, they have been - in the past, OAT was much more about control and morality, and it was very much like 'if you do this, then that happens' - it was probably more characterized by punishment. Uh, reward and punishment back then - in the past, you know. That mentality still lingers, and it affects those who have been in OAT for a long time more than those who enter today (pause). So they probably interpret or perceive it as a greater challenge, those who have experienced this. Conveying that we do this based on medical safety reasons and not control is a challenge, but we obviously have a need for control as well, right? It's part of our mandate too. [P4]

From the older generation, I experience a greater caution in engaging with OAT because many are quite content with being somewhat detached from the OAT system and have very little contact with us. I believe that in the past, OAT was perceived more as a strict control regime, so those who have been in OAT for a long time are very satisfied with having minimal involvement with us. However, what I am experiencing now is that many of the older individuals are a bit surprised that OAT is more interested in helping them engage in activities, assisting them in connecting with frontline services and the welfare agency (NAV). We offer much more support than just medications. [P9]

We can see practitioners here describing how negative experiences of a more punitive approach to OAT are carried into contemporary interactions, creating an extra layer of difficulty when communicating decisions taken on medical grounds that may be interpreted by older patients as being about control/punishment for their own sake. This is a specific challenge for older patients who have been in OAT since its early days when it was stricter, less flexible, and more control oriented. There was also an acknowledgement that contemporary control in OAT is also experienced as a challenge:

[I]n many cases, those over 50 have been in OAT for a long time, and still experience being stuck in this, in a way, control regime, right, they experience it as a continued lack of trust, at least if you have been in OAT for 20-30 years, they still have to take urine tests, they still have to ask for permission to bring medicine on vacation, right? […] They probably feel - many still feel a bit trapped in such a system then, and you can understand that. [P9]

This experience of being ‘trapped’ was also raised in separate patient interviews. The experience of being subject to urine testing and other control/safety measures for between 20 and 30 years is likely to impact negatively on both self-image and sense of agency. Given these findings and the findings on stability above, we see potential for a specific age-informed treatment model for this patient group.

#### Interactions outside specialist OAT

Practitioners’ experiences of cooperation with municipal-level services, welfare services and other primary or specialised healthcare varied widely from very positive to highly negative. Home nursing care was raised as a useful, quality-of-life enhancing service for older patients who required a higher level of follow-up and/or had mobility problems that prevented in-person collection of medicines:

Yes, so I think that in those municipalities where a high proportion receive home nursing care, it works very well. They pick up on a lot of things and can help in relation to contact with the GP or hospital ward and things like that. [P6]

Well, there are several of them who have provided feedback that they are treated really well, some of them have home nursing every day, which provides a sense of security in their everyday lives. […] it's that sense of calmness and predictability, and also the fact that when they are met by those who have been with them for a long time, a relationship forms as well. [P3]

Practitioners here describe both the direct influence of home nursing care in terms of the calmness, predictability and security these relationships can provide, but also the more indirect functions in terms of helping navigate the rest of the health and welfare system, including contact with GPs or secondary care services. Regarding GPs, there was again variation reported:

There's a huge difference between general practitioners, for example. Some allocate a whole hour for appointments, ask good questions, follow up on what they need to, and you can see that they care about the patient. On the other hand, other general practitioners constantly look at the clock, have only set aside 20 minutes, don't really have time to be present, and don't grasp what you're talking about. They seem disconnected, in a way. [P5]

An experience of a disconnected or even uninterested GP is likely to connect with older patients’ previous negative experiences with authority figures and as such have more impact than it would on someone without such baggage. On the other hand, an experience of care and commitment from a GP can be important both in terms of what it symbolises as well as in terms of the quality of the treatment/diagnosis itself. At the municipal level, poor attitudes and lack of service provision were also repeatedly highlighted as an issue. For example:

We have examples of very sick OAT patients, who receive help for everything other than their OAT medication, which they must collect themselves. We have rather grotesque examples from some municipalities, where things have been very, very difficult. […] Regarding short-term stays in nursing homes, they have rotating spots, which I think has worked very well for those who need and want it. I also think the nurses do a fantastic job with these patients. However, in other municipalities, as I mentioned before, attitudes can be very rigid and challenging, and there is as such stigmatisation that as a result, our patients do not receive the help they should. They face much higher expectations in terms of what they should be able to manage themselves and they [in the municipalities] say that it is for the patients’ own good. Some statements made by certain individuals are really jaw-dropping. [P6]

I think they have a moral problem. I think they have a principle - they are against OAT. And they've been quite clear about that, historically as well, some of these municipalities. So the municipal administration, they say they're against OAT, and that's why they don't want to distribute OAT medication. They also justify it with safety considerations and so on, but they are happy to distribute other medication to the same patients, so it doesn't quite add up. There aren't many, but we have some municipalities where it's a problem. [P4]

These excerpts reinforce previous observations about how negative attitudes about OAT patients impact directly on service provision. This becomes particularly acute as patients age, and they require—and have a legal right to receive—more support from municipal-level services. The first excerpt here also links back to previous discussion on living accommodation, in that having flexibility regarding the availability of short-term nursing home placements—a municipal service—is described as beneficial for some patients. Other participants relate an experience that appears rooted less in culture problems but in the technocratic challenge of delivering joined-up services:

It's also a frustrating thing, it's changed, actually - it could be that I took my eye off the ball a bit, but in the last three years, maybe four, then there has been a gradual change in that the municipality has, the large municipalities have slipped into an idea that now [the patients] are anchored in the specialist health service, therefore it is the specialist health service that must coordinate all services, whereas before it was a much more clear three-party collaboration, or four-party collaboration, with the GP, the municipality, the specialist health service/OAT and the patient. We've been to several of them, it's not specific just to this group, but several have been met with "yes, no, we're not offering anything, you have to sort it out". So, we are more and more alone with them. [P3]

This type of struggle to avoid service-delivery silos is hardly unique to either OAT or Norway. But when we are considering multiply-marginalised people who may struggle with issues of shame and self-worth, and with navigating an increasingly complex and remote systems of healthcare and welfare, such silo-driven provision has particularly acute implications.

In summary, this subsection has shown how positive interactions with home nursing care can support OAT and increase quality of life, but also that practitioners see significant cultural-attitudinal and geographical variation in how OAT patients are met by other elements of the welfare state outside OAT.

## Concluding discussion

The aim of this article was to analyse how OAT practitioners perceive the treatment older patients receive both in OAT and more broadly. In overall terms, our findings sit between the more optimistic perspectives on older OAT patients (e.g. [15]) and the more pessimistic (e.g. [33]), in that whilst practitioners view older patients (age 50 and above) as relatively stable in terms of lifestyle, substance use, and living arrangements, they also recognize significant challenges regarding cognitive impairment, loneliness/isolation, and comorbidities. Practitioners also recognised the duality of their own relationships with older OAT patients and of the impact that appropriate/substandard interactions with other parts of the system could have.

We identify a new form of cohort effect, whereby older patients’ experience of the earlier, stricter, and less flexible implementation of OAT in Norway is carried into contemporary interactions. Given that these experiences may have comprised being excluded from treatment for substance use, significant imposition of control/safety measures like urine testing^3^ (in 2003, 77% of patients underwent urine testing at least weekly [19], p. 11)), and breaches of relationships with treatment personnel, it is understandable that practitioners describe older patients as approaching their current treatment with caution.

A second scholarly contribution is regarding the increasing challenge of cognitive impairment in older Norwegian OAT patients, which impacts not only on how specialist OAT should deliver its services, but also has consequences for primary and social care delivery at the municipal level in Norway. The high degree of delegation down to Norway’s 357 local municipalities, which range in population from 215 to 717,000, creates challenges for structuring appropriate levels of services to people with complex needs. We recommend therefore initiating a pilot study on supported accommodation for vulnerable older patients.

The challenges that practitioners described have important implications for practice. Here are five key implications:

- **Individualized care**: Care provision should be based on patients' needs and functioning, avoiding age-based thresholds that may not reflect their actual health status. Adequate services should be available at both municipal/primary care and specialist/secondary care levels, requiring appropriately staffed supported accommodations to cater to the growing number of older OAT patients.
- **Managing cognitive impairment**: Problems with cognitive impairment necessitate adjustments in service provision and daily practice. Helping patients manage their medicines safely becomes increasingly crucial. Municipalities must fulfil their legal responsibilities in delivering OAT medicines via home care nursing to those who require it. A pilot study on supported accommodation that provides appropriate care for patients with cognitive impairment and dementia is also needed.
- **Addressing negative attitudes**: Negative attitudes towards OAT patients, particularly if from professional service providers in some municipalities, require higher scrutiny from central authorities. Both the central authorities in the municipalities as well as the Norwegian Directorate of Health should evaluate and, where appropriate, address lacking service provision and professionalism in municipal-level provision for OAT patients.
- **Tailored treatment model**: A new treatment model (in accordance with [40]), specifically designed for older OAT patients should consider their additional complexities, including cognitive impairment, the nature of support offered by OAT, and implement lighter-touch control/medical safety measures, when appropriate.
- **Enhancing communication**: When re-engaging with older patients who have had little contact with specialist OAT, dialogue about the treatment's evolution over time is helpful to ensure awareness of OAT's emphasis on flexibility and user involvement, especially focusing on the needs experienced by older OAT patients.

The study has a number of limitations. One is the small sample size, in that we conducted fine-grained analysis of interviews with 10 practitioners. However, observations made align with findings from larger-scale survey data (e.g. [27, 29]. In terms of the identity of the sample, there may have been self-selection bias, with practitioners most interested in quality treatment volunteering to participate. It is also possible that practitioners either actively seek to present their institutions in a positive way or are unconsciously biased towards identifying failings elsewhere, thereby placing greater responsibility for problems with other parts of the system or with patients (see [40] for patient perspectives). There was, though, hardly a Panglossian take on specialist OAT services from our participants. Finally, our decision not to present demographic information prevents making observations based on participants’ age/gender.

Further research is needed in several areas: the previously mentioned pilot study on supported accommodation for vulnerable older patients, register-based research on multiple-prescribing, and a survey-based study on how municipalities work with OAT patients are all relevant. Overall, addressing the challenges posed by an aging cohort of OAT patients requires a targeted and planned approach, focusing on cognitive functioning, adapted accommodation, and managing somatic and mental comorbidities. This approach ensures that OAT and other services meet this patient group with respect and dignity throughout their life course, and that the systems aligns to the current change in patient characteristics.

## Data Availability

The interview data used in this study contain sensitive personal information. As such, the transcripts are not publicly available. All excerpts used in the study have been de-identified. For queries on this please contact John Todd-Kvam, the corresponding author.

## Footnotes

1 We follow terminology recommendations from the National Institute on Drug Abuse.

2 Regarding the relationship between substance use and criminalisation, Norway scores well overall in the Global Drug Policy Index (1st place out of 30 countries and 74/100 points), albeit that where it scores weakest is in the category “Proportionality of criminal justice system response”, including scoring poorly regarding imprisonment for non-violent drug offences.

3 A recent review article highlights a lack of evidence for the effectiveness of urine drug screening on patient/community health outcomes [26].

## References

[1] Ayres RM, Eveson L, Ingram J, Telfer M (2010). Treatment experience and needs of older drug users in Bristol, UK. J Substance Use.

[2] Baldacchino A, Armanyous M, Balfour DJK, Humphris G, Matthews K (2017). Neuropsychological functioning and chronic methadone use: a systematic review and meta-analysis. Neurosci Biobehav Rev.

[3] Bamberg M, Georgakopoulou A (2008). Small stories as a new perspective in narrative and identity analysis. Text Talk.

[4] Bjørnestad ED, Vederhus J-K, Clausen T (2022). High smoking and low cessation rates among patients in treatment for opioid and other substance use disorders. BMC Psychiatry.

[5] Bukten A, Skurtveit S, Waal H, Clausen T (2014). Factors associated with dropout among patients in opioid maintenance treatment (OMT) and predictors of re-entry. A national registry-based study. Addict Behav.

[6] Carew AM, Comiskey C (2018). Treatment for opioid use and outcomes in older adults: a systematic literature review. Drug Alcohol Depend.

[7] Carlsen S-EL, Gaulen Z, Alpers SE, Fjaereide M (2019). Beyond medication: Life situation of older patients in opioid maintenance treatment. Addict ResTheory.

[8] Clausen T, Anchersen K, Waal H (2008). Mortality prior to, during and after opioid maintenance treatment (OMT): a national prospective cross-registry study. Drug Alcohol Depend.

[9] Conner KO, Rosen D (2008). “You're Nothing But a Junkie”: Multiple Experiences of Stigma in an Aging Methadone Maintenance Population. J Soc Work Pract Addict.

[10] Dursteler-MacFarland KM, Vogel M, Wiesbeck GA, Petitjean SA (2011). There is no age limit for methadone: a retrospective cohort study. Subst Abuse Treat Prev Policy.

[11] Eide D, Skurtveit S, Clausen T, Hesse M, Mravčík V, Nechanská B, Rolova G, Thylstrup B, Tjagvad C, Seid AK, Odsbu I, Gabrhelík R (2023). Cause-specific mortality among patients in treatment for opioid use disorder in multiple settings: a prospective comparative cohort Study. Eur Addict Res.

[12] European Monitoring Centre for Drugs and Drug Addiction. European Drug Report 2023: Trends and Developments. 2023. https://www.emcdda.europa.eu/publications/european-drug-report/2023_en

[13] Fareed A, Casarella J, Amar R, Vayalapalli S, Drexler K (2009). Benefits of retention in methadone maintenance and chronic medical conditions as risk factors for premature death among older heroin addicts. J Psychiatr Pract.

[14] Fereday J, Muir-Cochrane E (2006). Demonstrating rigor using thematic analysis: a hybrid approach of inductive and deductive coding and theme development. Int J Qual Methods.

[15] Firoz S, Carlson G (2004). Characteristics and treatment outcome of older methadone-maintenance patients. Am J Geriatr Psychiatry.

[16] Gaulen Z, Alpers SE, Carlsen SL, Nesvag S (2017). Health and social issues among older patients in opioid maintenance treatment in Norway. Nordisk Alkohol Nark.

[17] Gossop M, Stewart D, Treacy S, Marsden J (2002). A prospective study of mortality among drug misusers during a 4-year period after seeking treatment. Addiction.

[18] Han B, Polydorou S, Ferris R, Blaum CS, Ross S, McNeely J (2015). Demographic trends of adults in New York city opioid treatment programs–an aging population. Subst Use Misuse.

[19] Hansen MB, Kornør H, Waal H (2004) STATUSRAPPORT FOR PASIENTER I LEGEMIDDELASSISTERT REHABILITERING 2002–2003. In SKR-rapport nr 4/2004 (pp. 81).

[20] Helsedirektoratet. Nasjonal faglig retningslinje for legemiddelassistert rehabilitering (LAR) ved opioidavhengighet [nettdokument]. Helsedirektoratet. 2022. https://www.helsedirektoratet.no/retningslinjer/behandling-ved-opioidavhengighet. Accessed 6 Oct 2022

[21] Hser YI, Hoffman V, Grella CE, Anglin MD (2001). A 33-year follow-up of narcotics addicts. Arch Gen Psychiatry.

[22] Irani E (2018). The Use of Videoconferencing for Qualitative Interviewing: Opportunities, Challenges, and Considerations. Clin Nurs Res.

[23] Lintzeris N, Rivas C, Monds LA, Leung S, Withall A, Draper B (2016). Substance use, health status and service utilisation of older clients attending specialist drug and alcohol services. Drug Alcohol Rev.

[24] Luoma JB, Twohig MP, Waltz T, Hayes SC, Roget N, Padilla M, Fisher G (2007). An investigation of stigma in individuals receiving treatment for substance abuse. Addict Behav.

[25] Mayock P, Butler S (2021). “I’m always hiding and ducking and diving”: the stigma of growing older on methadone. Drugs.

[26] McEachern J, Adye-White L, Priest KC, Moss E, Gorfinkel L, Wood E, Cullen W, Klimas J (2019). Lacking evidence for the association between frequent urine drug screening and health outcomes of persons on opioid agonist therapy. Int J Drug Policy.

[27] Medved D, Clausen T, Bukten A, Bjornestad R, Muller AE (2020). Large and non-specific somatic disease burdens among ageing, long-term opioid maintenance treatment patients. Subst Abuse Treat Prev Policy.

[28] Muller AE, Skurtveit S, Clausen T (2017). Building abstinent networks is an important resource in improving quality of life. Drug Alcohol Depend.

[29] Nesse L, Lobmaier P, Skeie I, Lillevold PH, Clausen T (2023) Statusrapport 2022 - Første år med nye LAR-retningslinjer.

[30] Neumann CB, Neumann IB. Power, culture and situated research methodology: autobiography, field, text (1st ed. 2018. ed.). Springer. 2018

[31] Nyhagen HT, Waal H (2017). Den aldrende LAR pasienten. Nordisk Alkohol Nark.

[32] Roe B, Beynon C, Pickering L, Duffy P (2010). Experiences of drug use and ageing: health, quality of life, relationship and service implications. J Adv Nurs.

[33] Rosen D, Hunsaker A, Albert SM, Cornelius JR, Reynolds CF (2011). Characteristics and consequences of heroin use among older adults in the United States: a review of the literature, treatment implications, and recommendations for further research. Addict Behav.

[34] Rosen D, Smith ML, Reynolds CF (2008). The prevalence of mental and physical health disorders among older methadone patients. Am J Geriatr Psychiatry.

[35] Skeie I, Clausen T, Hjemsæter AJ, Landheim AS, Monsbakken B, Thoresen M, Waal H (2022). Mortality, causes of death, and predictors of death among patients on and off opioid agonist treatment: results from a 19-year cohort study. Eur Addict Res.

[36] Smith J, Osborn M, Smith JA (2007). Interpretative phenomenological analysis. Qualitative psychology: a practical guide to research methods.

[37] Subramanian N. Treatment and care for older drug users, European Monitoring Centre for Drugs and Drug Addiction (EMCDDA), 2010. Luxembourg: Publications Office of the European Union, 2010. Ir J Psychol Med, 2014;28(4):234–234. 10.1017/s0790966700011770. ISBN 978-92-9168-453-3

[38] Todd-Kvam J (2020). Probation practice, desistance and the penal field in Norway. Criminol Crim Just.

[39] Todd-Kvam J, Todd-Kvam M (2022). Talking good: analysing narratives of desistance in Norway. Br J Criminol.

[40] Todd-Kvam J, Sugahara G, Muller AE, Clausen T. Quality of life whilst ageing in Opioid Agonist Treatment: a narrative analysis. BMC Public Health **(Under review)** 10.1186/s12889-025-22438-4 PMC11948769 40141009

[41] Waal H, Bussesund K, Clausen T, Lillevold PH. Kjønn og alder i LAR. In: SERAF RAPPORT 1/2018. Oslo: SERAF. 2018

[42] Zolopa C, Høj SB, Minoyan N, Bruneau J, Makarenko I, Larney S (2022). Ageing and older people who use illicit opioids, cocaine or methamphetamine: a scoping review and literature map. Addiction.

